# Congenital Short Bowel Syndrome With Annular Pancreas Presenting as Neonatal Intestinal Obstruction

**DOI:** 10.7759/cureus.31802

**Published:** 2022-11-22

**Authors:** Deepti Naik, Santosh K Mahalik, Akash B Pati

**Affiliations:** 1 Pediatric Surgery, All India Institute of Medical Sciences (AIIMS) Bhubaneswar, Bhubaneswar, IND

**Keywords:** total parenteral nutrition (tpn), annular pancreas, neonatal intestinal obstruction, short bowel syndrome, congenital

## Abstract

Congenital short bowel syndrome (CSBS) is a very rare gastrointestinal anomaly of unknown etiology. We report a case of a six-week-old male with CSBS who presented with features of intestinal obstruction and failure to thrive. The abdominal radiograph was suggestive of a central gasless abdomen, and a provisional diagnosis of malrotation of the gut with volvulus was considered. On exploration, the duodenum was hugely dilated with annular pancreas, which was not obstructing the duodenum. The length of the intestine was 20 cm from the duodenojejunal junction to the ileocecal junction. Duodenal web was ruled out. Total parenteral nutrition (TNP) was started postoperatively. Early and long-term parenteral nutrition and referral to specialist centers with intestinal rehabilitation programs have improved the overall outcome; however, the challenges are entirely different in developing countries.

## Introduction

Congenital short bowel syndrome (CSBS) is a rare gastrointestinal anomaly characterized by a substantial reduction of small bowel length in the intrauterine period as compared to normal individuals limiting enteral digestion and absorption of nutrients. These patients present within a few days after birth with bilious vomiting, diarrhea, and failure to thrive. The prevalence is less than one in 1,000,000 births, and until recently, less than 65 cases have been reported in English literature [1,2]. This article describes a rare case of a six-week-old male with CSBS with an annular pancreas, who presented with features of intestinal obstruction. The association of the annular pancreas was previously not reported in English literature.

## Case presentation

A six-week-old male child presented with bilious vomiting for two weeks and failure to thrive. He was the second born from a non-consanguineous marriage, late preterm, appropriate for gestational age baby, and born by normal vaginal delivery with a birth weight of 2.4 kg. The antenatal ultrasonography was within normal limit. The baby cried immediately after birth, passed urine and meconium within 24 hours, and started on breastfeeds. He was detected as COVID-19-positive on the first day of life and was discharged with advice for home quarantine. He was feeding inadequately from then onward, however, and was passing urine and stool.

Since the third week of life, he had bilious vomiting and was managed conservatively elsewhere, with nasogastric aspiration and intravenous fluids. At referral, the child weighed 1.9 kg (smaller than the fifth percentile), and his length was 55 cm (within the 25th percentile). He appeared clinically well with no dysmorphic features, rashes, jaundice, or edema. On examination, his abdomen was soft, without any distension or organomegaly.

**Figure 1:**
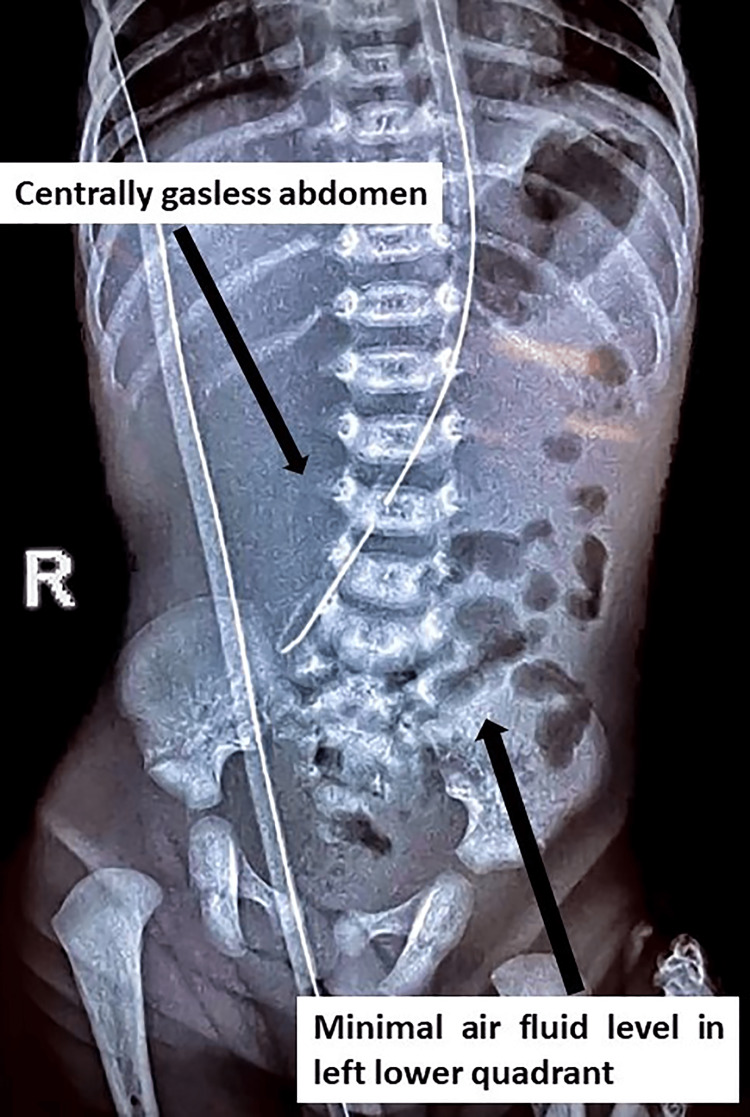
Abdominal radiograph showing a central gasless abdomen and sparsely scattered minimal air-filled bowel loops toward the left lower quadrant of the abdomen

Laboratory studies demonstrated hemoglobin of 17.3 g/dL, white blood cell count was 8,860/cmm with normal differential counts, and platelet count was 456,000/mcL. Serum electrolytes, creatinine, and bicarbonate levels were within normal limits. The serum calcium level was low, for which correction was given. An abdominal radiograph showed fundic gas, a central gasless abdomen, and sparsely scattered minimal air-filled bowel loops toward the left lower quadrant of the abdomen (Figure 1).

**Figure 2:**
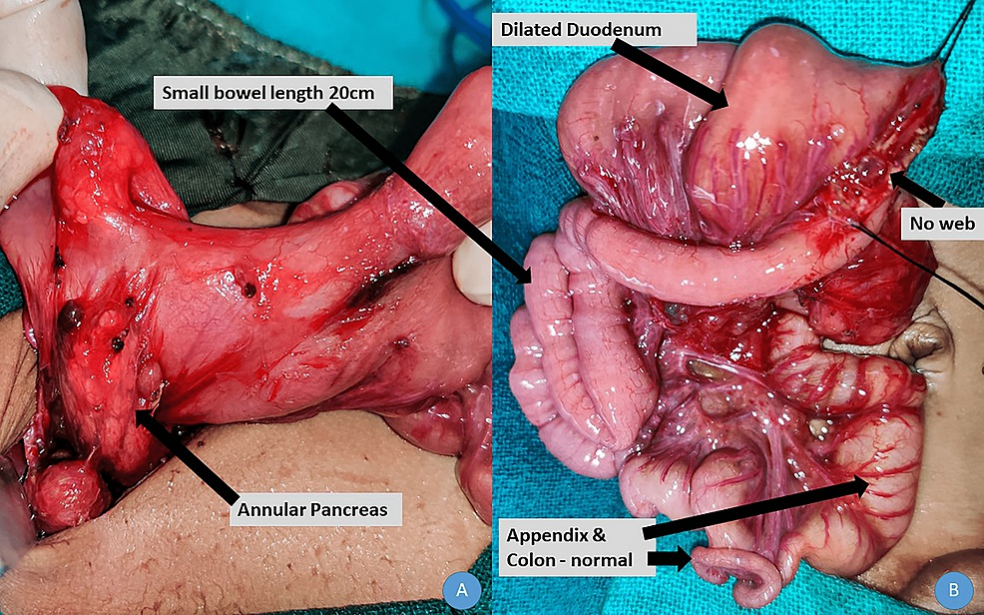
Operative photograph depicting the dilated duodenum, the presence of annular pancreas, short small intestine, and normal colon

Ultrasound was suggestive of collapsed small bowel loops, and the rest appears to be within normal limits. Because of no improvement with conservative management, bilious nasogastric aspirate, and a central gasless abdomen, a provisional diagnosis of malrotation of the midgut with volvulus was considered. Exploratory laparotomy was performed via a supraumbilical right transverse incision. The stomach was normally distended, and the duodenum was grossly dilated until the duodenojejunal junction. There were some atypical bands between the stomach and the colon. The ventral part of the pancreas encroached the wall of the duodenum without encircling it, thereby not causing duodenal obstruction. The small bowel was 20 cm from the duodenojejunal junction to the ileocecal junction. The appendix and entire colon were normal (Figure 2).

A longitudinal duodenal enterotomy was performed at the duodenojejunal junction, and duodenal web was ruled out. A diamond-shaped duodenojejunal anastomosis was performed, and the abdomen was closed. The baby was monitored postoperatively in neonatal intensive care unit (NICU) and started on total parental nutrition. The grave prognosis of the condition was explained to the parents and caregivers and the need for long-term total parental nutrition and the complications associated with it. They were also explained the need for genetic testing of the baby and the parents. However, they were not financially ready to bear the cost of the treatment and unfortunately left against medical advice on postoperative day 4.

## Discussion

The exact etiology of CSBS is unknown; however, the most accepted theory is the vascular insult during the embryonic period. CSBS is found to be related to an autosomal recessive mutation in coxsackie and adenovirus receptor-like membrane protein (CLMP) or X-linked recessive mutation in filamin A (FLNA) [2]. Only males develop disease associated with X-linked FLNA and have multiple congenital anomalies associated. Familial occurrence among siblings was reported, and consanguinity was also found to have some association [1]. In our case, the patient was a first-born male and a product of non-consanguineous marriage, and we were unable to identify any definite genetic etiology as the parents took the child against medical advice.

Negri et al., recently in a case series on CSBS including 61 patients over 50 years, concluded that the most common presentation was abdominal distension (96.2%) followed by failure to thrive (77.3%) and diarrhea (37%). The associated anomalies reported were bowel adhesion, bands, pyloric hypertrophy, persistent ductus arteriosus, central nervous system malformation, mesenterium commune, and the absence of the appendix [3]. In our case, the child presented with bilious vomiting and feeding intolerance with failure to thrive without any abdominal distension. The abdominal X-ray was mostly gasless with sparse gas, which raised the clinical suspicion of intestinal malrotation. As the child presented late, we did not perform any contrast study and decided to go for an emergency laparotomy. Our case was unique as it was associated with a massively dilated duodenum with an annular pancreas.

Annular pancreas is also a rare congenital anomaly characterized by a complete or partial ring of pancreatic tissue encircling the second part of the duodenum [4]. Non-bilious vomiting is commonly seen in patients with annular pancreas, and its diagnosis is difficult preoperatively. In our case, there was partial encircling of the pancreatic tissue without any duodenal compression, and the cause of duodenal dilatation could not be ascertained as we had ruled out duodenal web; however, it may be due to segmental dilatation following vascular ischemia, which has also resulted in short bowel in this case.

There is no consensus on the definition of small bowel syndrome in relation to the length of the remaining intestine. The prognosis of CSBS highly depends on the length and function of the remaining segment of the small bowel. Less than 40 cm traditionally requires therapy in most centers for survival [5]. Recently, the survival rate of CSBS has increased dramatically with multidisciplinary nutritional support management. The principle is to promote gut adaptation with the ultimate aim of achieving enteral autonomy while supporting growth and nutrition with parenteral nutrition. There are three phases of nutritional management, first being early restoration of fluid and electrolyte followed by macro- and micronutrient to promote growth and development through total parenteral nutrition (TPN) and lastly promoting gut adaptation and establishing early enteral feed [6].

Parenteral nutrition has been a huge support in these patients; however, the rate of complications such as sepsis and liver failure is very high. Bowel-lengthening procedures such as serial transverse enteroplasty (STEP) and plication of dilated duodenum have been used in some centers, and small bowel transplantation is considered the last resort due to overall poor survival [7]. The unavailability of multidisciplinary nutritional support centers and affordability are serious concerns in patients from low socioeconomic status in developing countries such as India, which was the case for our patient.

## Conclusions

CSBS is a rare condition that presents with bile-stained vomitus, failure to thrive, and diarrhea. A contrast study may give a clue; however, the definite diagnosis is always at laparotomy. Annular pancreas presents as a proximal intestinal obstruction. Imaging studies have a limitation to detect the short bowel syndrome in neonates when there is an associated proximal bowel obstruction, which precludes gas to traverse beyond the obstruction. Hence, emergency physicians will have an on-table surprise to detect the CSBS along with annular pancreas. Early and long-term parenteral nutrition and referral to specialist centers with intestinal rehabilitation programs have improved the overall outcome of this rare condition worldwide. However, the challenges are even more in the developing part of the world.
